# Real-World Retrospective Study into the Effects of Oral Semaglutide (As a Switchover or Add-On Therapy) in Type 2 Diabetes

**DOI:** 10.3390/jcm12186052

**Published:** 2023-09-19

**Authors:** Riccardo Candido, Sara Gaiotti, Fabiola Giudici, Barbara Toffoli, Federica De Luca, Valerio Velardi, Alessandra Petrucco, Chiara Gottardi, Elena Manca, Iris Buda, Bruno Fabris, Stella Bernardi

**Affiliations:** 1 Department of Medical Surgical and Health Sciences, University of Trieste, 34149 Trieste, Italy; riccardo.candido@units.it (R.C.); gaiotti.sara@gmail.com (S.G.); fabiola.giudici@cro.it (F.G.); btoffoli@units.it (B.T.); federica.deluca9612@gmail.com (F.D.L.); valerio.velardi@gmail.com (V.V.); b.fabris@fmc.units.it (B.F.); 2 SC Patologie Diabetiche, ASUGI (Azienda Sanitaria Universitaria Giuliano Isontina), 34128 Trieste, Italy; alessandra.petrucco@asugi.sanita.fvg.it (A.P.); chiara.gottardi@asugi.sanita.fvg.it (C.G.); elena.manca@asugi.sanita.fvg.it (E.M.); iris.buda@asugi.sanita.fvg.it (I.B.); 3 SS Endocrinologia Medicina Clinica, ASUGI (Azienda Sanitaria Universitaria Giuliano Isontina), 34128 Trieste, Italy

**Keywords:** type 2 diabetes mellitus, oral semaglutide, GLP-1 RA, switch, HbA1c, BMI, real world

## Abstract

(1) Background: Oral semaglutide represents the first oral GLP-1 RA approved for the treatment of type 2 diabetes mellitus (T2DM). This real-world retrospective study aimed at evaluating its effectiveness and tolerability in the treatment of patients with T2DM when patients switched from a glucose-lowering agent to it and when it was added to the usual therapy. (2) Methods: Adult patients with T2DM taking oral semaglutide and followed in the ASUGI Diabetes Center were identified with the use of electronic medical records between October 2022 and May 2023. (3) Results: A total of 129 patients were recruited. The median follow-up was 6 months. Be it as a switchover or as an add-on therapy, oral semaglutide significantly reduced HbA1c and BMI. Switching from DPPIV inhibitors to oral semaglutide was associated with a significant reduction in HbA1c and BMI, switching from SGLT2 inhibitors was associated with a significant reduction in HbA1c, and switching from sulphonylureas was associated with a significant reduction in BMI. The median HbA1c change was associated with baseline HbA1c. SBP significantly decreased in the add-on group. Oral semaglutide was well tolerated. (4) Conclusions: This study shows that in the real-world setting, oral semaglutide is effective and safe as a switchover or as an add-on therapy for the treatment of T2DM.

## 1. Introduction

Semaglutide is a human glucagon-like peptide-1 receptor agonist (GLP-1 RA). GLP-1 RAs are an established class of glucose-lowering medication that significantly decrease fasting and post-prandial levels of blood glucose, glycated hemoglobin, and body weight in patients with type 2 diabetes mellitus (T2DM) [1]. In addition, regardless of structural homology, they reduce the risk of chronic diabetic complications, including major adverse cardiovascular events, all-cause mortality, hospital admission for heart failure, and worsening kidney function in patients with T2DM [2]. Interestingly, GLP-1RAs seem to reduce the rate of cardiovascular and renal endpoints even beyond their ability to lower glucose concentrations [3,4], and this could be ascribed to body weight reduction and to the amelioration of blood pressure and lipid profile [5,6,7], as well as to their anti-inflammatory activity [4,8,9]. In other words, GLP-1 RAs address cardiometabolic risk factors comprehensively [1,3].

Semaglutide is the first GLP-1 RA that can be taken orally. This relies on an advanced pharmaceutical technology to ensure its absorption and effectiveness when it is ingested: semaglutide is combined with SNAC (sodium N-[8-(2-hydroxybenzoyl) amino] caprylate) to protect the peptide from enzymatic degradation in the stomach and to increase its absorption [10]. Compared with injectable GLP-1 RAs, the oral formulation is more convenient and has the advantage of greater patient acceptability [11].

Oral semaglutide efficacy and safety were evaluated in the peptide-innovation-for-early-diabetes-retreatment (PIONEER) randomized controlled trials (RCTs), which showed a significant improvement of glycemic control, weight reduction, and cardiovascular risk biomarkers (e.g., blood pressure, lipids, abdominal adiposity, and inflammation), with non-inferiority to placebo in terms of cardiovascular safety [12,13,14,15,16,17,18,19,20,21]. Nevertheless, only a few studies into the real-world use of this drug have been published so far [22,23,24], and it remains to be fully established what the effects are of this drug in routine clinical practice, not only when oral semaglutide is added to other medication but also when patients switch from a glucose-lowering agent to it.

Based on these premises, this real-world retrospective study aimed at (i) describing the characteristics (e.g., age, sex, and diabetes duration) and therapeutic regimen (e.g., oral monotherapy, dual therapy, or injectable therapy) of patients starting oral semaglutide and (ii) evaluating its effectiveness and tolerability in the treatment of T2DM as a switchover and as an add-on therapy. The primary endpoint was HbA1c change, and secondary endpoints were body mass index (BMI) and blood pressure changes from baseline to last follow-up. Adverse events were also recorded.

## 2. Materials and Methods

### 2.1. Study Design

This was an observational retrospective study. Adult patients (age > 18 years) with T2DM taking oral semaglutide and followed in the ASUGI Diabetes Center were identified with the use of electronic medical records between October 2022 and May 2023. The ASUGI Diabetes Center (S.C. Patologie Diabetiche, ASUGI, Trieste, Italy) is an outpatient clinic for diabetic patients, which is part of a public University Hospital. The prescription of oral semaglutide was based on the principles of care and management of T2DM recommended by national [25,26] and international guidelines [27,28]. Exclusion criteria included (i) follow-up data not available; (ii) patients not taking prescribed medication; (iii) type 1 diabetes mellitus or gestational diabetes; (iv) not providing informed consent to participate in this study.

Data were collected with electronic healthcare records. They included demographic characteristics, disease duration, medication (withdrawn and/or associated, if any), final dose of oral semaglutide, and length of follow-up (from oral semaglutide introduction to last visit), as well as HbA1c, body weight, body mass index (BMI), systolic and diastolic blood pressure (SBP and DBP), frequency and cause of drug discontinuation, and frequency and type of adverse events. In general, BMI was calculated from dividing the patient’s weight in kilograms (kg) by their height in squared meters (m). Blood pressure was measured using an aneroid sphygmomanometer.

This study was conducted in accordance with the Declaration of Helsinki, and the protocol was approved by the Institutional Review Board of the University of Trieste, Trieste, Italy (CEUR 107_2022H).

### 2.2. Statistical Analysis

All statistical analyses were carried out in the R system for statistical computing (Ver 5.0; R development Core Team, The R Foundation, Vienna, Austria, 2018). Statistical significance was set at *p* < 0.05. The Shapiro–Wilk test was applied to quantitative variables to check for distribution normality. Quantitative variables were each reported as a median with IQR. Changes over time were reported as absolute differences (follow-up-value–baseline-value). Quantitative variables were compared by the Mann–Whitney test (and Kruskall–Wallis test) depending on the number of groups. Qualitative variables were reported as absolute frequencies and percentages and compared with the chi-squared test. Multivariate linear regression was used to explore the effects of several factors on the change over time of HbA1c, BMI, and SBP. Results are reported in terms of beta regression coefficients with 95% confidence intervals. As for the power calculation, based on [22], 34 patients (with baseline and follow-up data) are required to detect a mean HbA1c change of −0.9% (SD = 1.8), providing 80% power at a 5% level of significance (two-tailed paired Student’s *t*-test).

## 3. Results

### 3.1. Population Characteristics

A total of 129 patients were identified, and their characteristics are reported in Table 1. The median age was 72 years (IQR 66; 79), and 74/129 (57%) patients were men. Diabetes duration was 11 years (IQR 6; 22). The baseline median HbA1c was 7.2% (IQR 6.6; 8) and BMI was 28.8 kg/m^2^ (IQR 26.3; 32.8). Rybelsus was started at the dose of 3 mg for 4 weeks, and then—based on glucose control and gastrointestinal tolerability—it was increased up to the final dose of 7 mg in 77/129 patients (60%) and 14 mg in 38/129 patients (29%). The median follow-up after the introduction of oral semaglutide was 6 months (IQR 6; 12). In 70/129 (54%) patients, the last follow-up was 6 months after oral semaglutide introduction; in 37/129 (29%) patients, it was after 12 months; and in 22/129 (17%) patients, it was after 3 months.

In the majority of patients, oral semaglutide was prescribed in replacement of another glucose-lowering drug (SWITCH group, *n* = 99/129; 77%), while in the remaining ones (ADD-ON group, *n* = 30/129; 23%), it was added to the usual therapy. Be it in the SWITCH or ADD-ON group, a total of 122/129 (95%) patients were taking oral semaglutide in addition to other agents, while only 7/129 (5%) patients were taking oral semaglutide as a monotherapy. The agents to which it was added were metformin (84/129; 65%), SGLT2 inhibitors (68/129; 53%), insulin (36/129; 28%), sulphonylureas (17/129; 13%), and pioglitazone (11/129; 8.5%). The characteristics of the entire population, SWITCH, and ADD-ON groups (including the medication associated with oral semaglutide) are reported in Table 1.

### 3.2. HbA1c Change after Oral Semaglutide Introduction

#### 3.2.1. HbA1c Change in the Entire Population, SWITCH, and ADD-ON Groups

**Figure 1:**
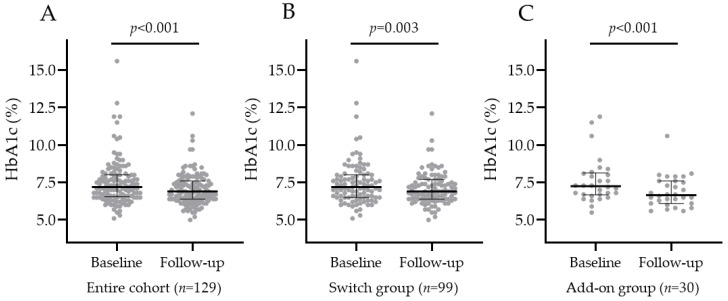
HbA1c in the entire cohort (**A**), SWITCH group (**B**), and ADD-ON group (**C**) before and after oral semaglutide introduction.

In the entire population, baseline HbA1c was 7.2% (IQR 6.6; 8), and oral semaglutide significantly decreased it to 6.9% (IQR 6.4; 7.6) at the last follow-up, as shown in Figure 1A. In the SWITCH group, HbA1c significantly decreased from 7.2% (IQR 6.5; 8.0) to 6.9% (IQR 6.4; 7.6), and the median HbA1c change was −0.3% (IQR −0.7; 0.3). In the ADD-ON group, HbA1c significantly decreased from 7.3% (IQR 6.7; 8.1) to 6.7% (IQR 6.2; 7.6), and the median HbA1c change was −0.4% (IQR −0.9; −0.1), as shown in Figure 1B,C. There was no significant difference in the median HbA1c percentage variation after oral semaglutide introduction between the SWITCH and ADD-ON groups (*p*-value = 0.12).

#### 3.2.2. HbA1c Change Based on the Type of Medication Switchover

**Figure 2:**
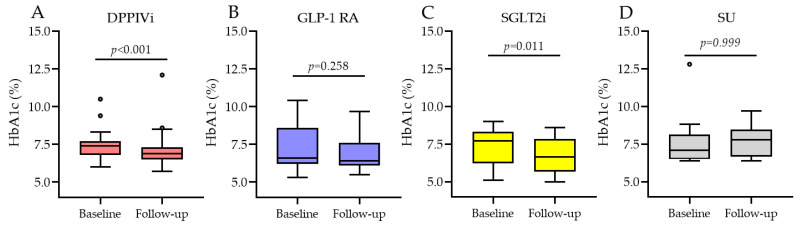
HbA1c before and after oral semaglutide introduction in the SWITCH subgroups. (**A**) is change from DPPIVi to oral semaglutide; (**B**) is change from injectable GLP1-RA to oral semaglutide; (**C**) is change from SGLT2i to oral semaglutide; (**D**) is change from sulphonylureas to oral semaglutide.

**Figure 3:**
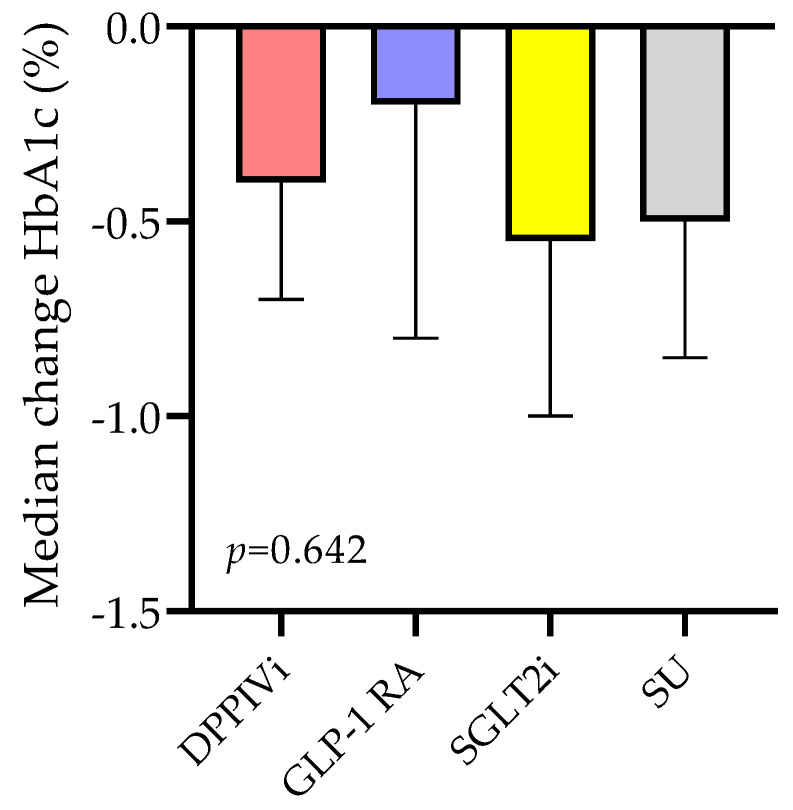
Median HbA1c change in the SWITCH subgroups.

The SWITCH group was divided into four subgroups based on the type of switchover: switch from dipeptidyl peptidase IV (DDPIV) inhibitors (DPPIVi, *n* = 35); switch from subcutaneous GLP-1 RA (GLP-1 RA, *n* = 15); switch from sodium-glucose co-trasporter-2 (SGLT2) inhibitors (SGLT2i, *n* = 12); and switch from sulphonylureas to oral semaglutide (SU, *n* = 9). As shown in Figure 2, in the DPPIVi–oral-semaglutide SWITCH subgroup, HbA1c significantly decreased from 7.4% (IQR 6.9; 7.7) to 6.9% (IQR 6.6; 7.3), *p*-value < 0.001, and the median HbA1c change was −0.4% (IQR −0.7; −0.2). In the subcutaneous GLP-1 RA–oral-semaglutide SWITCH subgroup, HbA1c went from 6.6% (IQR 6.3; 8.6) to 6.4% (IQR 6.2; 7.3), *p*-value = 0.26, and the median HbA1c change was −0.2% (IQR −0.6; 0.25). In the SGLT2i–oral-semaglutide SWITCH subgroup, HbA1c significantly decreased from 7.7% (IQR 6.2; 8.2) to 6.7% (IQR 6.0; 7.6), *p*-value = 0.01, and the median HbA1c change was −0.55% (IQR −0.8; −0.1). In the SU–oral-semaglutide SWITCH subgroup, HbA1c did not significantly change, *p*-value = 0.99, and the median HbA1c change was −0.5% (IQR −0.7; 1.4). Comparison between the median HbA1c changes in these four subgroups showed no significant differences between them (Figure 3), *p*-value = 0.64.

### 3.3. BMI and Body Weight Change after Oral Semaglutide Introduction

#### 3.3.1. BMI Change in the Entire Population, SWITCH, and ADD-ON Groups

**Figure 4:**
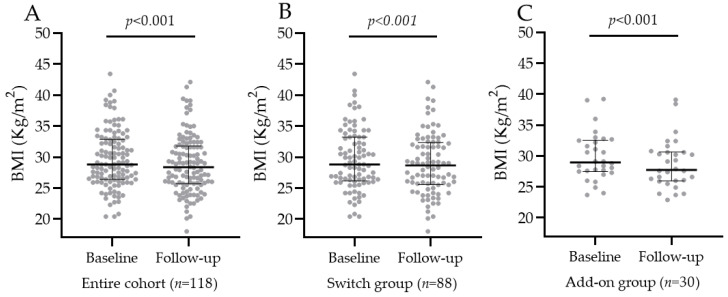
BMI in the entire cohort (**A**), SWITCH group (**B**), and ADD-ON group (**C**) before and after oral semaglutide introduction.

In 118/129 (91%) patients, BMI was recorded at baseline and at last follow-up. In the entire population, baseline BMI was 28.8 kg/m^2^ (IQR 26.4; 32.8), and oral semaglutide significantly decreased it to 28.35 kg/m^2^ (IQR 25.7; 31.7) at last follow-up, *p*-value < 0.001, as shown in Figure 4A. In the SWITCH group, BMI decreased from 28.8 kg/m^2^ (IQR 26.3; 33.2) to 28.7 kg/m^2^ (IQR 25.7; 32.3), and the median BMI change was –0.7 kg/m^2^ (IQR −1.23; 0). In the ADD-ON group, BMI decreased from 29 kg/m^2^ (IQR 27.5; 32.4) to 27.8 kg/m^2^ (IQR 26; 30.6), and the median BMI change was −1.15 kg/m^2^ (IQR −2.1; −0.63), as shown in Figure 4B,C. The introduction of oral semaglutide reduced BMI significantly more in the ADD-ON group vs. SWITCH group, *p*-value = 0.01.

#### 3.3.2. BMI Change Based on the Type of Medication Switchover

**Figure 5:**
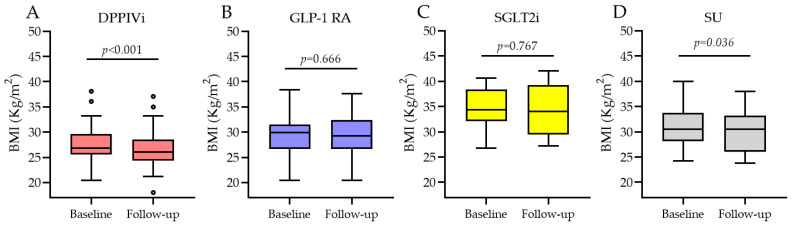
BMI before and after oral semaglutide introduction in the SWITCH subgroups. (**A**) is change from DPPIVi to oral semaglutide; (**B**) is change from injectable GLP1-RA to oral semaglutide; (**C**) is change from SGLT2i to oral semaglutide; (**D**) is change from sulphonylureas to oral semaglutide.

**Figure 6:**
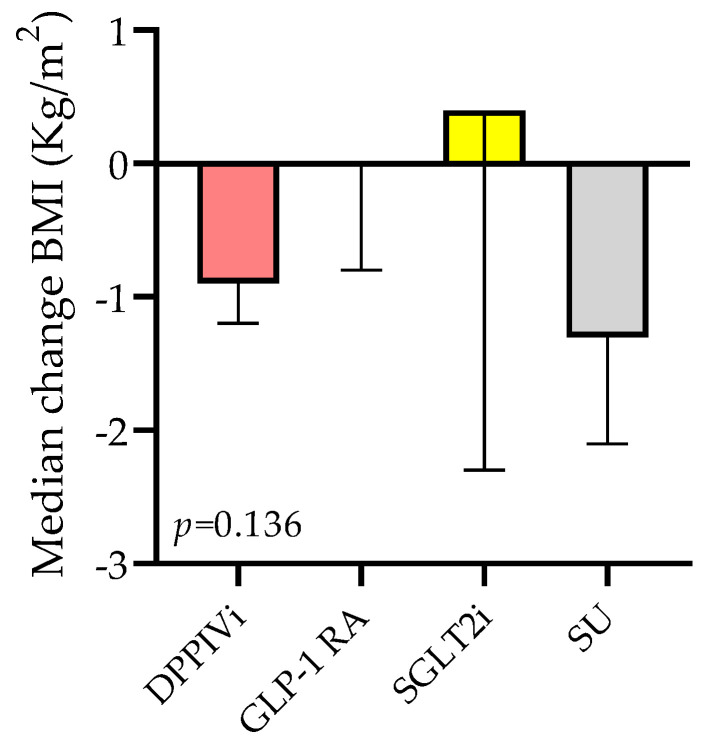
Median BMI change in the SWITCH subgroups.

When looking at the four SWITCH subgroups (Figure 5), in the DPPIVi–oral-semaglutide SWITCH subgroup, BMI significantly decreased from 26.8 kg/m^2^ (IQR 25.8; 29.3) to 26.1 kg/m^2^ (IQR 24.4; 28), *p*-value < 0.001, and the median BMI change was −0.9 kg/m^2^ (IQR −1.2; −0.4). Likewise, in the SU–oral-semaglutide SWITCH subgroup, BMI significantly decreased, and the median BMI change was −1.3 kg/m^2^ (IQR −2.05; −0.3). By contrast, BMI did not change in the switch from the subcutaneous GLP-1 RA to the oral semaglutide group (median change 0) and from SGLT2i to oral semaglutide group (median change 0.4). Comparison between the median BMI changes in these four subgroups showed high variability but no significant differences between them (Figure 6), *p*-value = 0.14.

#### 3.3.3. Body Weight Change after Oral Semaglutide Introduction

In 98/129 (76%) patients, body weight was recorded at baseline and at last follow-up. As shown in Table 2, body weight decreased by −2.0 kg (IQR −4.4; 0) in the entire population. Body weight decreased more in the ADD-ON than in the SWITCH group, i.e., −3.5 (−6.1; −1.9) vs. −2.0 kg (IQR −3; 0), respectively. When looking at the SWITCH subgroups, body weight decreased by −2.1 (−3.2; −1.08) in the DPPIVi–oral-semaglutide switchover; it remained unchanged in the GLP-1 RA–oral-semaglutide subgroup; it decreased by −3.0 (−6.0; 2.0) in the SGLT2i–oral-semaglutide subgroup; and it decreased by −3.0 (−6.0; −2.0) in the SU–oral-semaglutide subgroup (*p* = 0.02). In addition, as shown in Table 2, in the entire population, 27.6% of patients exhibited a body weight reduction ≥ 5%, and 6.1% of patients exhibited a body weight reduction ≥ 10%. There was a higher proportion of patients exhibiting weight loss ≥ 5% and ≥ 10% in the ADD-ON group. In particular, in the ADD-ON group, 41.4% of patients had a body weight loss ≥ 5%, and 16.7% had a body weight loss ≥ 10%, compared with 22.9% and 2.7% in the SWITCH group.

### 3.4. Blood Pressure Change after Oral Semaglutide Introduction

**Figure 7:**
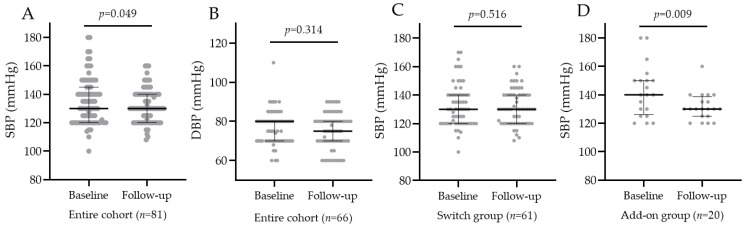
SBP and DBP in the entire cohort before and after oral semaglutide introduction (**A**,**B**). SBP in the SWITCH group (**C**) and ADD-ON group (**D**) before and after oral semaglutide introduction.

In 81/129 (63%) patients, blood pressure was recorded at baseline and at last follow-up. In the entire population, SBP significantly decreased after oral semaglutide introduction (*p*-value = 0.05), but DBP did not, as shown in Figure 7A,B. In the SWITCH group, SBP did not change, while in the ADD-ON group, SBP decreased from 140 (IQR 129; 150) to 130 (IQR 125; 136), and the median SBP change was −12.5 mmHg (IQR −20; 15), as shown in Figure 7C,D. The introduction of oral semaglutide reduced SBP significantly more in the ADD-ON group vs. SWITCH group, *p*-value = 0.01.

### 3.5. Multivariate Linear Regression Analyses

**Figure 8:**
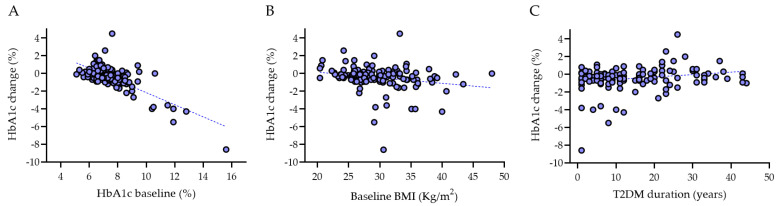
Univariate correlation between HbA1c change and baseline HbA1c (**A**), HbA1c change and BMI (**B**), as well as between HbA1c change and T2DM duration (**C**).

Table 3, Table 4 and Table 5 show the results of multivariate linear regression analyses. HbA1c change was independently associated with both baseline HbA1c and baseline BMI as well as with T2DM duration (Figure 8). The SBP change was independently associated with T2DM duration as well as with taking oral semaglutide as a switchover or as an add-on therapy. No significant associations were observed for BMI change.

### 3.6. Oral Semaglutide Discontinuation and Adverse Events

Oral semaglutide was discontinued in 13/129 (10%) patients. A total of 10/129 (7.75%) patients reported adverse events; in 3/129 (2.32%) patients, the medication was discontinued and changed to insulin because it did not achieve glucose control. Adverse events that led to oral semaglutide discontinuation included gastrointestinal symptoms (8/129; 6.2%), itching (1/129; 0.8%), and symptomatic hypoglycemia (1/129; 0.8%). Overall, a total of 4/129 (3%) patients experienced hypoglycemic episodes; these were all patients who were taking oral semaglutide in addition to insulin.

## 4. Discussion

The extraordinary success of GLP-1 RAs in T2DM treatment is based on the properties of the incretin hormone GLP-1, which increases the insulin secretory response to an oral glucose load, suppresses glucagon secretion, decelerates gastric emptying, and reduces food seeking [3,29]. Oral semaglutide, which is a combination of the GLP-1 RA semaglutide and the absorption-enhancer sodium N-amino caprylate, represents the first oral GLP-1 RA. The efficacy and safety of oral semaglutide have been evaluated in the PIONEER randomized controlled trials (RCTs) [12,13,14,15,16,17,18,19,20,21], which showed a significant improvement of glycemic control, body weight, and cardiovascular risk biomarkers, including blood pressure, lipids, abdominal adiposity, and inflammation, with non-inferiority to placebo in terms of cardiovascular safety.

Our real-world retrospective study shows that oral semaglutide significantly reduced HbA1c and body weight in T2DM patients not only when it was given as an add-on therapy but also when it was prescribed as a switchover from a different glucose-lowering drug. Multivariate linear regression analysis showed that HbA1c change was independently associated with both HbA1c and BMI at baseline (i.e., the greater baseline HbA1c and BMI were, the greater HbA1c decreased), and HbA1c change was independently associated with T2DM duration (patients with longer history of T2DM had lower HbA1c reduction). When comparing the SWITCH with the ADD-ON groups, there were no differences in terms of median HbA1c change, which were −0.3% and −0.4%, respectively. By contrast, BMI was reduced significantly more in the ADD-ON (−1.15 kg/m^2^) vs. the SWITCH group (−0.7 kg/m^2^). This is likely due to the association with other drugs reducing BMI in the ADD-ON group, e.g., SGLT2i [30] were prescribed to 67% of patients in the ADD-ON group vs. 48.5% of patients in the SWITCH group, although this difference did not reach statistical significance (*p*-value = 0.08). It is also possible that the previous use of an injectable GLP-1 RA in some patients of the SWITCH group had already helped reduce BMI. Not surprisingly, possibly for the same reasons, also body weight change and the percentage of patients with body weight loss ≥ 5% and ≥10% were greater in the ADD-ON group compared with the SWITCH group.

Overall, oral semaglutide significantly reduced HbA1c level when it was given as a switchover medication (median HbA1c change −0.3%). There was no significant difference between the switchover subgroups in terms of HbA1c change. When looking at HbA1c before and after oral semaglutide introduction in each subgroup, the switchovers associated with significant HbA1c changes were DPPIVi–oral-semaglutide (*p* < 0.001) and SGLT2i–oral-semaglutide (*p*-value = 0.01). Our study showed that when switching from DPPIVi to oral semaglutide, the median HbA1c change was −0.4%. This finding is consistent with the results of the PIONEER 3 and 7 trials [14,18,31]. The PIONEER 3 trial showed that the 7 and 14 mg oral semaglutide were superior to sitagliptin in reducing HbA1c after 26 weeks [14]. The PIONEER 7 trial showed that oral semaglutide with flexible dose adjustment was superior to sitagliptin in terms of the odds of achieving on HbA1c of less than 7% [18]. In addition, an open-label extension of the PIONEER 7 trial showed that switching from sitagliptin to oral semaglutide helped more patients achieve HbA1c targets and greater reductions in body weight [31]. As for the effect of the switchover from SGLT2i to oral semaglutide, our results are in line with the PIONEER 2 trial [13], showing that oral semaglutide was superior to empagliflozin in reducing HbA1c, but not body weight, after 26 weeks [13]. Interestingly, in this trial [13], the estimated treatment difference between SGLT2i and oral semaglutide was −0.5% in terms of HbA1c change at week 26, which is in line with our data showing that the switchover from SGLT2i to oral semaglutide was associated with a median HbA1c change of −0.55% [13].

Likewise, BMI significantly decreased when oral semaglutide was given as a switchover medication (−0.7 kg/m^2^). The degree of BMI change varied across the switchover subgroups, being the greatest in the DPPIVi–oral-semaglutide and the SU–oral-semaglutide SWITCH subgroups. This finding is consistent with the results of the PIONEER 3 and 7 trials [14,18,31]. Interestingly, also the switch from sulphonylureas to oral semaglutide was associated with a significant BMI reduction. It has to be noted that this is the first report of the effects of the switchover from sulphonylureas to oral semaglutide. Despite the small number of patients, our results show that in patients taking sulphonylureas, the switchover to oral semaglutide did not significantly change HbA1c but significantly reduced BMI, which supports the indication of prescribing glucose-lowering drugs with proven cardiovascular and renal benefits (such as GLP-1 RAs and SGLT2i) first, instead of sulphonylureas [32]. The striking reduction in the use of sulphonylureas observed in our study (only 13% of patients were taking it) reflects the recommendations of the Italian guidelines for the treatment of type 2 diabetes (which have both clinical and medico-legal implications), saying not to use and to de-prescribe this class of drugs [25,26].

SBP and DBP were recorded in half of the patients. In keeping with the results of the PIONEER 6 trial [17], our study shows that oral semaglutide reduced significantly SBP, but not DBP, in the entire cohort. Interestingly, we found that SBP changed significantly in the ADD-ON group (−12.5 mmHg) compared with the SWITCH group, where it remained unchanged. A recent real-world study has also reported that oral semaglutide significantly reduced SBP from 135 mmHg to 129 mmHg after 6 months when it was added to metformin and SGLT2i but did not have effects in patients who had undergone the switchover from a once-weekly subcutaneous GLP-1 RA to oral semaglutide [23]. It is likely that in our study, the greatest effects on SBP were seen in the ADD-ON group, not only because in the SWITCH group there were patients switching from subcutaneous to oral GLP-1RA but also because 67% of patients in the ADD-ON group were taking SGLT2i compared with 48.5% in the SWITCH group (*p*-value = 0.08). Multivariate linear regression analysis showed that the association of oral semaglutide with other glucose-lowering agents had a significant impact on SBP reduction, while patients with longer history of T2DM had lower SBP reduction.

Our data show that treatment with oral semaglutide was well tolerated, with a safety and tolerability profile consistent with the GLP-1 RA class [12]. In particular, gastrointestinal discomfort, nausea, and diarrhea were reported by 6.2% of patients, whereas hypoglycemic episodes were recorded in 3% of patients. It has to be noted that in our cohort, hypoglycemic events occurred in patients who were taking oral semaglutide in addition to insulin. Overall, our results are similar to the incidence of adverse events reported in the PIONEER 1 study [12], where gastrointestinal symptoms were the most frequent complaint, which was reported by a proportion of patients ranging from 5.1% to 16%, while hypoglycemic episodes were reported in 4.6% of patients. Based on their effectiveness and the safety and tolerability profile, as well as the convenience of the oral formulation, it is conceivable that GLP-1 RAs might be positioned earlier in T2DM management.

Finally, we discuss the strengths and limitations of this study. Observational studies can complement the information provided by RCTs, as they offer insights as to whether an intervention is effective and safe in day-to-day clinical practice in a more heterogeneous patient group [33]. The first strength of this study, which is one of the first observational real-world analyses on the effectiveness of oral semaglutide [22,23,24], is that it is the first study that has evaluated this drug in a population of patients that were 72 years old and who had baseline HbA1c of 7.2% and baseline BMI of 28.8 kg/m^2^. In other words, our study demonstrates that oral semaglutide was effective and safe in a population that was older and had lower baseline HbA1c and BMI compared not only with the few observational studies that have been published so far [22,23,24] but also with the PIONEER trials [12,13,14,15,16,19,31]. In the PIONEER trials, patients’ ages were between 55 and 70 years, baseline HbA1c was between 8% and 8.3%, and BMI was between 31–33 kg/m^2^ [12,13,14,15,16,18,19]. In the observational studies that are available, patients’ ages were between 58 and 63 years, baseline HbA1c was between 8.1% and 8.8%, and BMI was between 30.5–36.2 kg/m^2^ [22,23,24]. The second strength of our study is that it takes into account the effects of a switchover from different classes of glucose-lowering drugs (including DPPIVi, injectable GLP-1 RAs, SGLT2i, and SU) to oral semaglutide. However, the small sample size of these subgroups is a limitation of the present study, and therefore, the results of the subgroup analyses should be considered preliminary data.

## 5. Conclusions

This study is one of the first real-world analyses on the effectiveness of oral semaglutide as a switchover or as an add-on therapy for the treatment of T2DM. Here, we show that oral semaglutide was effective and safe in a T2DM population that was older and had lower baseline HbA1c and BMI compared with the PIONEER trials and the literature. These data and the convenience of the oral formulation might help position GLP-1 RAs earlier in T2DM management.

## Figures and Tables

**Table 1:** Population characteristics.

		All Patients (*n* = 129)	SWITCH Group (*n* = 99)	ADD-ON Group (*n* = 30)	*p*-Value
Age (years)		72 (66; 79)	73 (66.5; 79)	69.5 (62; 79)	0.31
Sex	M	74 (57%)	57 (58%)	17 (57%)	0.92
	F	55 (43%)	42 (42%)	13 (43%)	
Diabetes duration (years)		11 (6; 22)	12 (6; 22.5)	8 (6; 12)	0.12
Baseline HbA1c (%)		7.2 (6.6; 8)	7.2 (6.5; 8)	7.3 (6.7; 8.1)	0.75
Baseline BMI (kg/m^2^)		28.8 (26.3; 32.8)	28.8 (26.3; 33.2)	29 (27.5; 32.4)	0.55
Dose of oral semaglutide (mg)		7 (7; 14)	7 (7; 14)	7 (7; 14)	0.28
	3 mg	14 (11%)	13 (13%)	1 (3%)	0.31
	7 mg	77 (60%)	58 (59%)	19 (63%)	
	14 mg	38 (29%)	28 (28%)	10 (33%)	
Last follow-up (months)		6 (6; 12)	6.9 (6.5; 8)	6.65 (6.7; 8.1)	0.27
Monotherapy	Yes	7 (5%)	7 (7%)	0 (0%)	0.13
	No	122 (95%)	92 (93%)	30 (100%)	
Therapy w metformin	Yes	84 (65%)	64 (65%)	20 (67%)	0.83
	No	45 (35%)	35 (35%)	10 (33%)	
Therapy w SGLT2i	Yes	68 (53%)	48 (48.5%)	20 (67%)	0.08
	No	61 (47%)	51 (51.5%)	10 (33%)	
Therapy w insulin	Yes	36 (28%)	24 (24%)	12 (40%)	0.09
	No	93 (72%)	75 (76%)	18 (60%)	
Therapy w sulphonylureas	Yes	17 (13%)	15 (15%)	2 (7%)	0.23
	No	112 (87%)	84 (85%)	28 (93%)	
Therapy w pioglitazone	Yes	11 (8.5%)	10 (10%)	1 (3%)	0.24
	No	118 (91.5%)	89 (90%)	29 (97%)	

The *p*-value refers to the SWITCH vs. ADD-ON group. The last follow-up is for the last follow-up after oral semaglutide introduction; SGLT2i is for sodium-glucose co-trasporter-2 inhibitors; w is for with.

**Table 2:** Body weight change.

Body Weight Loss	Entire Cohort (*n* = 98)	SWITCH Group (*n* = 74)	ADD-ON Group (*n* = 24)	*p*-Value
Median absolute change (IQR)	−2.0 (−4.4, 0)	−2.0 (−3.0, 0)	−3.5 (−6.1, −1.9)	0.007 ^a^
≥5%	27 (27.6%)	17 (22.9%)	10 (41.4%)	0.074 ^b^
≥10%	6 (6.1%)	2 (2.7%)	4 (16.7%)	0.013 ^b^

^a,b^ Comparison between SWITCH and ADD-ON groups; ^a^ Mann–Whitney test for independent data; ^b^ chi-squared test.

**Table 3:** Multivariate linear regression model for HbA1c change.

Variable	Beta	95% CI	*p*-Value
Age (years)	0.00	−0.02, 0.02	0.98
Sex: male vs. female	−0.07	−0.40, 0.26	0.67
Baseline HbA1c (%)	−0.70	−0.82, −0.58	<0.001
Baseline BMI (kg/m^2^)	−0.04	−0.08, −0.01	0.02
T2DM duration	0.02	0.00, 0.04	0.02
Dose of oral semaglutide (mg)	0.02	−0.03, 0.06	0.49
Last follow-up (months)	−0.02	−0.08, 0.03	0.44
Monotherapy vs. association	−0.30	−1.1, 0.50	0.56
SWITCH vs. ADD-ON group	0.25	−0.14, 0.65	0.21
Insulin (yes vs. no)	0.21	−0.22, 0.63	0.33
SGLT2 (yes vs. no)	0.12	−0.23, 0.47	0.51
R^2^ = 0.625			

**Table 4:** Multivariate linear regression model for BMI change.

Variable	Beta	95% CI	*p*-Value
Age (years)	−0.02	−0.04, 0.01	0.16
Sex: male vs. female	0.31	−0.19, 0.80	0.22
Baseline HbA1c (%)	−0.06	−0.23, 0.11	0.50
Baseline BMI (kg/m^2^)	−0.05	−0.11, 0.01	0.085
T2DM duration	−0.01	−0.04, 0.01	0.24
Dose of oral semaglutide (mg)	−0.03	−0.09, 0.04	0.42
Last follow-up (months)	−0.07	−0.16, 0.02	0.11
Monotherapy vs. association	0.02	−1.23, 1.28	0.97
SWITCH vs. ADD-ON group	0.53	−0.03, 1.08	0.06
Insulin (yes vs. no)	0.55	−0.07, 1.17	0.08
SGLT2 (yes vs. no)	−0.17	−0.70, 0.36	0.52
R^2^ = 0.149; AIC = 407			

**Table 5:** Multivariate linear regression model for SBP change.

Variable	Beta	95% CI	*p*-Value
Age (years)	0.07	−0.28, 0.42	0.695
Sex: male vs. female	3.2	−4.0, 10	0.38
Baseline HbA1c (%)	−0.70	−3.3, 1.9	0.59
Baseline BMI (kg/m^2^)	−0.63	−1.6, 0.30	0.18
T2DM duration	0.38	0.04, 0.72	0.03
Dose of oral semaglutide (mg)	0.17	−0.79, 1.1	0.72
Last follow-up (months)	0.54	−0.81, 1.9	0.43
Monotherapy vs. association	23	5.5, 40	0.01
SWITCH vs. ADD-ON group	5.8	−2.5, 14	0.17
Insulin (yes vs. no)	−0.78	−9.8, 8.2	0.86
SGLT2 (yes vs. no)	1.4	−6.0, 8.8	0.71
R^2^ = 0.268; AIC = 680			

## Data Availability

The raw data supporting the conclusions of this article will be made available by the authors upon reasonable request.
